# Trochisci Calami

**Published:** 1862-06

**Authors:** 


TROCHISCI CALAMI.—A very agreeable preparation for cases of flatulency and dyspepsia. ℞.—White sugar, six ounces; fluid extract of calamus, one fluid ounce; oil of fennel and oil of anise seed, of each twenty minims. Rub them together, and with mucilage of tragacanth form a mass, which is to be divided into one hundred and sixty lozenges.—*Med. and Surg. Reporter.*

